# Radiation-Induced Sarcoma following Prolonged Coronary Stent Placement

**DOI:** 10.1155/2018/2903801

**Published:** 2018-08-05

**Authors:** Eric J. Vick, Christopher T. Clark, James M. Lewis

**Affiliations:** ^1^College of Medicine, The University of Tennessee Health Science Center, Memphis, TN, USA; ^2^Department of Pathology, University of Tennessee Medical Center, Knoxville, TN, USA; ^3^Department of Surgical Oncology, University of Tennessee Medical Center, Knoxville, TN, USA

## Abstract

Radiation exposure for the average coronary stent placement varies based on a number of factors but typically amounts to 6–11 mSv per patient (compared to 3 mSv background). As with all procedures which utilize radiation, there is an inherent risk of genetic mutation and the possible development of malignancy. Here, we present the case of a 75-year-old male who presented with an exophytic mass on his back following prolonged coronary catheterization with a radiation burn seven years prior. Biopsy of the lesion revealed the mass was consistent with an undifferentiated pleomorphic sarcoma emanating from the site of the radiation burn. After staging studies demonstrated no evidence of metastatic disease, radical excision with negative margins was performed. This case demonstrates that despite the rarity of radiation injury, each incidence necessitates strict monitoring of radiation exposure and continual follow-up due to the risk of malignancy.

## 1. Introduction

Undifferentiated pleomorphic sarcomas (UPS), previously known as a pleomorphic malignant fibrous histiocytoma, is a diagnosis of exclusion. It is classified based on lack of any discernable cell lineage, at most sharing characteristics with mesenchymal stem cells and representing the third most common type of sarcoma found in general and the most common radiation-induced sarcoma (RIS) [1–3]. Presentation of sporadic UPS is widely varied in location, as cases have been documented in the stomach [4], vocal fold [5], scrotum [6], and among others. These tumors almost exclusively overlap with areas of radiation exposure in cases of RIS. The prognosis of radiation-induced sarcomas is associated with poorer clinical outcomes and higher recurrence rates as compared to sporadic lesions [7]. In the case of our patient, the likely etiology of this phenomenon is associated with the unique chromosomal aberrations and genetic mutations associated with RIS, including duplication and fusion of *MYC* [8] as well as the many factors which may delay a timely diagnosis. One component of the multimodal treatment of RIS may be the use of radiotherapy, which has proved one of the most effective treatments to date [9].

## 2. Case Report

**Figure 1:**
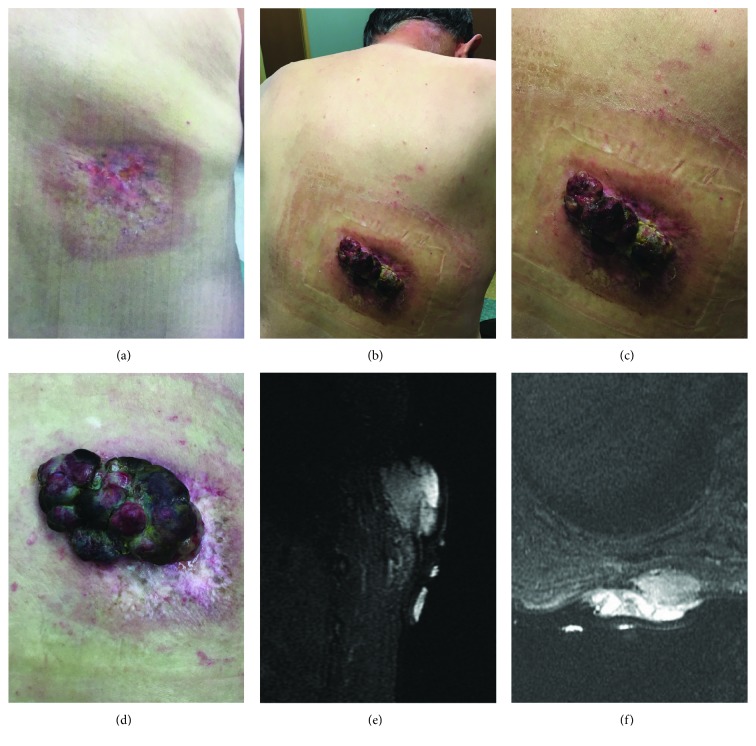
Gross imaging of the patient's mass: gross images of the area of ulceration which developed following cardiac catherization photographed in 2014 (a) show no signs of growth. In 2017, a mass (b) demonstrating outward growth and the area of the radiation injury following a year of close observation with a superimposed image of the same (c) mass was obviously exophytic and purulent measuring 5 × 3 cm at the time. The mass was photographed again prior to surgery two months later (d) in addition to MRI imaging shown with sagittal (e) and axial STIR (f) imaging demonstrating T1 signaling slightly intense to muscle and heterogeneous T2 signaling with avid contrast enhancement at the level of the posterior T10 rib. Both images fail to show evidence of muscular or bony invasion.

We present the case of a 75-year-old male whom initially presented to our service with a mass on his right upper back. The patient's medical history included coronary artery disease and hyperlipidemia. Further history revealed he had received (2) stents in the past and undergone multiple heart catheterizations (up to seven) approximately seven years prior at an outside facility. During his last catheterization and stent procedure, which took over 4 hours (approx. 33 mSv), he developed a radiation-induced injury to his right back around the T10 dermatome (Figure 1(a)). During the following years, the burn was closely followed at an outside facility, and the area developed a chronic nonhealing ulcer which continued to evolve over time eventually growing outward from his back and developing a foul odor on his presentation to our facility in 2017. He reported increasing pain at the periphery of the tumor margin. Up to a year prior to presentation, the area was flat and biopsies revealed no evidence of malignancy. Upon presentation to us, however, the physical exam demonstrated a fungating mass approximately 5 × 9 cm with central purulent necrosis and induration surrounding the periphery (Figures 1(b)–1(d)). No additional nodularity or adenopathy was found on physical exam initially. At this time, punch biopsies demonstrated undifferentiated pleomorphic sarcoma along with frankly necrotic debris.

Initial staging showed only localized disease by MRI with no evidence of metastasis by CT scan (Figures 1(e) and 1(f)). Approximately one month following diagnosis, the patient underwent a radical excision with a 20 × 9 cm elliptical excision of the site. In this case, the patient chose human dermal matrix reconstruction, as primary closure was not possible due to the size of the site. Our patient did not want an additional wound of autologous skin procurement.

**Figure 2:**
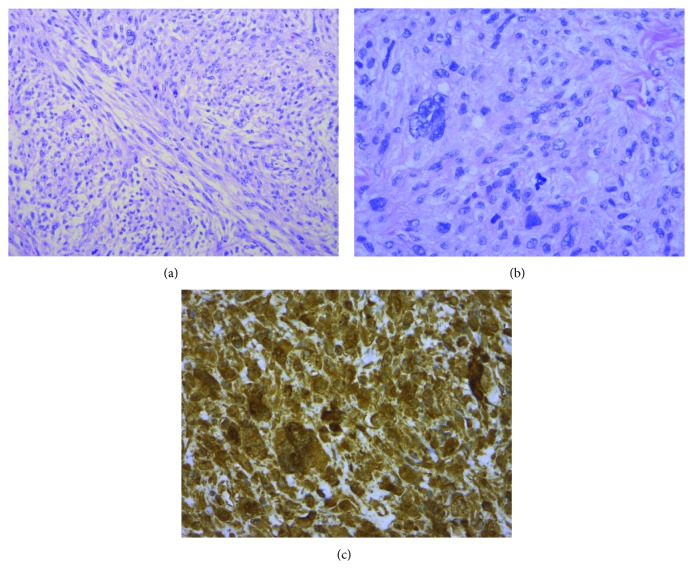
Undifferentiated pleomorphic sarcoma on microscopic analysis: H&E section (200x magnification) showing cellular spindle cell neoplasm with fascicular growth pattern (a). H&E section (400x magnification) showing pleomorphic vesicular nuclei with irregular nuclear contours and mitotic activity (b). Immunohistochemical stain (400x magnification) for vimentin strongly positive within neoplastic cells (c).

Pathology of histologic sections revealed a highly cellular spindle cell neoplasm. Focal areas of necrosis comprised less than 50% of sampled tissue. Mitotic figures were readily evident (17 per 10 high-power microscopic fields), including atypical forms. Marked pleomorphism was present with vesicular nuclei, irregular nuclear contours, and scattered prominent nucleoli (Figures 2(a) and 2(b)). The immunohistochemical staining pattern was supportive of sarcoma. The neoplastic cells are positive for CD68 and vimentin (Figure 2(c)). The neoplastic cells were negative for markers of melanocytic origin (S100 protein, sox10, and melan A), epithelial origin (pancytokeratin AE1/AE3), vascular origin (CD31), neural origin (S100 protein), and muscle origin (smooth muscle actin, myoD1, and desmin). MDM2 gene amplification by FISH testing was negative. Overall histologic and immunohistochemical staining features were those of undifferentiated pleomorphic sarcoma (pleomorphic malignant fibrous histiocytoma). The final pathology of the mass demonstrated a grade 3 undifferentiated pleomorphic sarcoma-staged pT2a. All margins were negative for malignancy obtaining an R0 resection. Furthermore, there was no evidence of angiolymphatic invasion. After discussion by our institutional multidisciplinary tumor board, adjuvant therapy was not recommended. The patient's postsurgical course was essentially benign with the exception of not unexpected wound healing issues. The patient is under continued observation with clinical follow-up in accordance with NCCN guidelines. Follow-up CT in October 2018 demonstrated pulmonary metastases, and he passed away from disease in June 2018.

## 3. Discussion

Cardiac catheterizations are exceptionally common in the US, with over four million such procedures conducted per year [10]. During these procedures, there is a documented risk following radiation exposure, especially when considering procedures which extend beyond 1 hour using fluoroscopy. This includes the possibility of a radiation burn like the one experienced by our patient. The radiation dose needed to cause a radiation burn varies and is likely underreported due to a delay in onset relative to the dose and associated environmental factors but is approximately 2 Sv [11]. The onset of the burn varies and may take a week or much longer to manifest. In higher doses, effects such as erythema, ulceration, and dermal atrophy will predominate [12]. Our patient described a similar phenomenon, which began as erythema in the week following his NSTEMI, which evolved into an ulcer over time.

In a cardiac catheterization, the priority is focused on the immediacy of the cardiac issue at hand. The prospect of enhanced radiation exposure may go unnoticed due to an obvious immediate risk-benefit ratio. However, enhanced radiation exposure has long-term effects for both staff and patients. One study of 859 people found that the dosage necessary to cause skin damage was exceeded for up to 22% of cardiac ablation procedure in adults and that the mean time of fluoroscopy was 53 min. Together, the authors estimate this would result in up to 4000 excess fatal malignancies all together [13]. Radiation has a stochastic effect meaning that any dose of radiation may lead to the development of malignancy, though higher doses having an increasingly linear dose-effect relationship [14]. Despite these findings, statistical models do not show evidence that cardiac catheterization leads to the development of malignancy. However, these models did not stratify those who had received a radiation-induced injury compared to those who did not [15]. In the case of our patient, even background radiation may have caused the development of a sarcoma given an ideal environment, but the UPS which developed in his burn site is a RIS known to develop at the site of radiation exposures.

Radiation-induced sarcomas (RIS), while rare, are a known complication. In most cases, RIS is the result of radiotherapy for cancer treatment, but any source of dose-dependent radiation may be sufficient to lead to the development of RIS. Radiation-induced malignancies may encompass any subtype of sarcoma, most commonly osteosarcoma and UPS. They may also lead to the development of malignancies, including lymphoma, leukemia, and solid tumors from other germ layers [16, 17]. In all cases, the increase in ionizing radiation leads to an increase in single- and double-stranded DNA breaks and eventually contributes to the aberrant replication of cell lines. Sarcomas arising from the mesenchymal origin represent one the highest proportion of radiation-induced cancers, presumably due to a large amount of actively dividing tissue over a large surface area. In a follow-up study of atomic bomb survivors, radiation of 1 Gy was found to double the risk of soft tissue sarcoma development. The five-year survival for these patients was also reduced, which may be attributable to the amount and type of mutations including replication of c-MYC found in this group of malignancies [8, 18]. Latency in this and other studies is also worth mentioning, as the mean delay from exposure to the diagnosis of RIS was 37 years. This delay contrasts with our case, which occurred observably over a period of seven years. The patient's older age of exposure may play some role in the acceleration of RIS development, but no conclusive study has been conducted. *U* is unique due to a high degree of dedifferentiation and aggressive potential and resistance to therapy with the possible exception of radiotherapy [7, 19, 20].

Patients with radiation injury may require close follow-up to monitor tissue repair and address malignant potential. This is the first case to our knowledge of RIS as a sequela of a cardiac catheterization procedure or involving c-arm-induced radiation exposure. In the case of our patient, his UPS was discovered early in the course of the disease and had yet to penetrate through the chest wall during his initial treatment. The development of his UPS following cardiac catheterization seven years prior represents a known phenomenon for RIS, but the first case to our knowledge of RIS following this procedure. It is our belief that while this represents a rare incident, it does represent a need for vigilance to observe patients following a cardiac catheterization. Patients as well as catheterization lab staff are at high risk in these situations to encounter significant radiation exposure, and the exposure to radiation is variable [21]. Some patient groups are already receiving more than the 2 mSv/year recommended by the National Council on Radiation Protection, recommended for radiation workers [22]. More institutional safety standards are necessary, as the mortality of the group from cancer is as high as 1 in 245. In addition, the increasing trend toward lower doses of radiation represents a positive measure both for patients and for lab staff. We are only beginning to appreciate the effects of <100 mGy (roughly equivalent to 100 mSv in living tissue) doses of radiation in long-term effects, and further study is needed both into the incidence of radiation injury following radiologic procedures and malignancy development in these cases.
